# Stretchable Hydrophobic Surfaces and Self-Cleaning Applications

**DOI:** 10.1038/s41598-019-50982-8

**Published:** 2019-10-11

**Authors:** Bekir Sami Yilbas, Ghassan Hassan, Hussain Al-Qahtani, Naser Al-Aqeeli, Abdullah Al-Sharafi, Abdulrahman S. Al-Merbati, Turki N. Baroud, Johnny Adukwu Ebaika Adukwu

**Affiliations:** 1 Department of Mechanical Engineering, King Fahd University of Petroleum and Minerals (KFUPM), Dhahran, 31261 Saudi Arabia; 2 Center of Research Excellence in Renewable Energy (CoRE-RE), King Fahd University of Petroleum and Minerals (KFUPM), Dhahran, 31261 Saudi Arabia; 3 Senior Researcher at K.A.CARE Energy Research & Innovation Center at Dhahran, Dhahran, Saudi Arabia; 4 Researcher at K.A.CARE Energy Research & Innovation Center at Dhahran, Dhahran, Saudi Arabia

**Keywords:** Mechanical engineering, Devices for energy harvesting

## Abstract

Hydrophobizing of stretchable elastomer surfaces is considered and the reversible behavior of the resulting surface wetting state is examined after stretching and relaxing the hydrophobized samples. The environmental dust are analyzed in terms of elemental constitutes and size, and the dust pinning on the hydrophobized surface is measured. The dust removal mechanisms, by the water droplets on the hydrophobized surface, are investigated. We demonstrated that deposition of functionalized nano-size silica units on the elastomer surface gives rise to hydrophobicity with 135° ± 3° contact angle and low hysteresis of 3° ± 1°. Stretching hydrophobized elastomer surface by 50% (length) reduces the contact angle to 122° ± 3° and enhances the hysteresis to 6° ± 1°. However, relaxing the stretched sample causes exchanging surface wetting state reversibly. Water droplet rolling and sliding can clean the dusty hydrophobized surface almost 95% (mass ratio of the dust particles removed). Droplet puddling causes striations like structures along the droplet path and close examination of the few residues of the dust reveals that the droplet takes away considerably large amount of dust from surface.

## Introduction

Surface cleaning from dusts remains important for sustaining the efficiency and output power of photovoltaic panels and concentrated solar troughs. Several cleaning processes have been used for cleaning of the surfaces. Some of these methods involves with high cost, such as water jet cleaning^1,2^, or excessive compressor power, such as air jet blowing^3^. Self-cleaning of surfaces is one of the cost effective processes for removing the settled particles. In general, self-cleaning of surface relies on: (i) reduction of adhesion of the settled particles on the surface, and (ii) increasing the mobility of the liquid droplets picking up the particles. To reduce the pinning of particles on surfaces, surface texture consisting of micro/nano pillars and low surface energy has to be created. Low surface energy and texture with micro/nano pillars alter the surface characteristic to a hydrophobic. Hence, mobility of the liquid droplets enhances via rolling and sliding while picking up the particles^4^. The reversible wetting state exchange remains fruitful for the self-cleaning applications such as droplet velocity can be controlled locally on the surface. Stretchable hydrophobic surfaces can vary the surface morphology and modify the surface wetting characteristics with varying contact angle. Although surface can remain hydrophobic, the alteration of the surface wetting state influences the dynamic motion of the droplet. The water droplet can be used for self-cleaning of surface^4^; however, changing the hydrophobic wetting state of the surface modifies the self-cleaning characteristics in terms of the dust removal rate. Consequently, investigation of droplet mobility and dust removal from surfaces with stretchable and reversibly exchanging wetting states becomes essential.

The reversible exchange of the wetting state depends on several factors and some of these include the variable hierarchal distributed surface morphology and varying free energy over the surface. The surface treatment of the polymeric materials with hydrophobic wetting states can provide the reversible exchanging of the wetting state; in such case, exposure of oxygen plasma and subsequent thermal treatment of surfaces can possibly provide the reversible transition from hydrophobic to superhydrophobic states and vice versa^5^. Chemical treatment of surfaces allows reversible exchange of the wetting state such as use of dithiolane as a platform for reversible surface functionalization, which enables to provide switchable water and oil-wettability^6^. Magnetron spattering of smart thin films, such as V_2_O_5_ thin films, results in the wetting state change reversibly from hydrophobic to superhydrophobic^7^. Some surface coatings and surface texturing processes give rise to reversible exchange of wetting state, such as precursor vacuum plasma spray process via injecting Yb(NO_3_)_3_ solution into a plasma jet operated at low pressure results in hierarchal distributed surface texture with reversible wetting state^8^. Growing a smooth copper oxide thin film on surfaces at room temperature can also result in a reversible wettability transition on the film surface^9^. Forming a composite film with hierarchical distributed TiO_2_/SiO_2_ can also provide reversible exchange of the wetting state under the UV radiation^10^. The micro-nano porous textures with hierarchal morphology enable to result in reversible exchange of the wetting state. In this case, the porous layer on copper surface, which is produced by the deposition technique, demonstrates hydrophobic wetting state in air. However, the wetting state changes once the surface is immersed into water and the wetting state reverses once the porous texture dries^11^. Phase change material, such as n-octadecane, can be used to create a reversible exchange of the wetting state^12,13^; however, the reversible exchange of the wetting state is limited with the small temperature range at which the phase change material undergoes melting and solidification. The surface hydrophobicity can also maintain for the hierarchical wrinkle stretchable surfaces even after being repeatedly stretched^14,15^. The reversible hydrophobic surface characteristics allow the substrates used in many applications such as textile and sensors. In addition, highly stretchable and hydrophobic surfaces can open door for new approaches towards tunable photonic system design and self-cleaning applications^16^.

On the other hand, efficient cleaning of hydrophobic surfaces using the water droplets depends on the droplet dynamics on the surface. In this case, the surface wettability is highly related to the dust removal rate, which is particularly true for the droplets formed from the water condensate^17^. Moreover, water droplet rolling and sliding on hydrophobic surfaces becomes current interest in energy sector and self-cleaning applications. The liquid droplet dynamics is highly related to the wetting state of the surfaces and it influences thermal state of the surface via heat transport^18^. The micro-pore created at the inclined surface alters the droplet dynamics; hence, the liquid droplet removal rate could be altered by modifying the state of droplet pinning via surface inclination and alteration of the micro-pore structures^19^. The change of the dynamic contact angle of the droplet influences the droplet behavior on the surface and it has also influence on the liquid droplet removal from the channel^20^. The numerical simulation of droplet motion on the hydrophobic surfaces remains interest towards predicting dynamic characteristics of the droplets for various applications, such as in fuel cells^21,22^. In self-cleaning applications, droplet dynamics play a major role for dust removal from surfaces^4^. The transition period of the wetting length on the surface remains longer the dust cloaking period of the droplet liquid; in this case, the dust can be removed by the droplet. The wobbling of the rolling droplet, under the influence of the gravity, limits the size of the dust removal area from the hydrophobic surface^23^.

The dynamics of the water droplet (rolling/sliding) can vary on the stretchable hydrophobic surfaces. This is because of the texture morphology, which is modified along the stretching length of the hydrophobic surface. Hence, the hydrophobic surface stretching can influence the self-cleaning characteristics in terms of the dust removal rates and the area cleaned. Although self-cleaning of surfaces utilizing the water droplet rolling and sliding has been investigated previously^4,23^, the hydrophobic surface has almost homogeneous texture morphology and uniform surface free energy. Investigation of the effects of the varying surface texture morphology on the self-cleaning characteristics is left for the future study. Altering the surface texture morphology influences the droplet pinning and its puddle on the surface while modifying the combination of the droplet rolling and sliding mechanisms on the surface. In addition, the wetting state of the stretchable surfaces were investigated previously^14–16^, the influence of the stretchable surface on the water droplet dynamics was left for further investigations. In the present study, the texture feature of the stretchable surface of hydrophobic characteristics is presented and the droplet movement on the stretchable surface is studied. Environmental dust characteristics and the mechanisms of dust removal from the stretchable surface by the droplet are investigated. The dust are gathered locally in Dammam area of Saudi Arabia and the polyisoprene elastomer is used to create the stretchable hydrophobic surface.

## Experimental

Polyisoprene elastomer, which had the same basic chemical formula as the natural rubber, was used in the experiments. The properties of the elastomer are given in Table 1. The wetting state of the elastomer surface was assessed and the droplet contact angle was measured as 72°, which was hydrophilic. The hydrophilic wetting state of the elastomer sample surfaces was changed to hydrophobic wetting state introducing the nano-size functionalized silica units on the surface via deep coating method. The nano-size silica units were, first, synthesized and, later, functionalized adopting the method used in the early work^24^. During the synthesizing process, the mixture was prepared incorporating ethanol (14.2 mL), ultrapure water (1.2 mL), and ammonium hydroxide (24 mL) via string at 350 rpm for 15 minutes. The diluted tetraethyl orthosilicate (TOES) (1 mL TOES in 4 mL ethanol) was added to the mixture. After adding the diluted TEOS, the mixture was left for 20 minutes and, later, diluted TEOS (0.5 mL TEOS in 2 mL ethanol) was added to the mixture enriching TEOS concentration. The modifier silane was included in 3:4 molar ratio to the mixture. It was, then, mixed magnetically for 20 hours. The mixture solution was, then, centrifuged and, later, ethanol was added towards ensuring the removal of the reactants. The deep coating method was introduced dispersing the functionalized nano-silica units on the sample surface. The sample surfaces were, then, dried under the vacuum to warrant removing the solvent residues, via evaporation, on the coated surface. A mechanical assembly was developed to stretch the coated elastomer samples uniformly by 50% of the original sample size. It should be noted that increasing the stretch length more than 60% of the original sample length results in permanent damages, such as fractured and partially pealing, on the coating located on the elastomer surface; in which case, the wetting state could not be reversibly exchange via stretching and relaxing. A care is taken to maintain the reversible exchange of the wetting state on the sample surface. Hence, the stretching length is limited to 50% of the original length of the coated sample. The deep coating process provided almost uniform coating thickness of about 500 ± 30 nm. The scratch tests were carried out to assess the adhesion of the coating on to the elastomer surface. The average tangential force required to remove the coating from the surface was determined as 530 mN, which indicated the good bonding between the coating and the elastomer surface. Fourier transform infrared spectroscopy (FTIR) was conducted using Nicolet Nexus 670 Spectrometer. The FTIR data revealed that the bending modes of Si-O-Si took place at 800 cm^−1^. The peak occurring at 1400 cm^−1^ was related to the bending modes of C-H of the organic residues^25^.

**Table 1:** Properties of polyisoprene elastomer used in the analysis.

Thermal Conductivity (W/mK)	Density (kg/m^3^)	Thermal Expansion Coefficient (1/K)	Specific Heat (J/kgK)	Elongation at break (%)	Tensile Strength MPa)	Upper Working temperature (K)
0.29	930	5 × 10^−5^	450	200–800	28	473

The coated elastomer sample surfaces prior to and after stretching were investigated using JEOL 6460 scanning electron microscope with high magnification (300000×), 3-D optic microscope, and atomic force microscope (AFM, NanoMagnetics Instruments). The wetting state of the sample surfaces were determined incorporating the contact angle measurements, which were assessed through adopting the technique introduced earlier^26^. The droplet tracking during rolling and sliding on the hydrophobized elastomer sample surfaces with and without dust was realized using the high speed data recording (SpeedSense 9040). The dust was collected from the Dammam area in Saudi Arabia during the period of three months. During the dust collection, the soft brushes were used to remove dust from the surfaces of the photovoltaic panels and the dust was kept in an airtight sample bottles. The dust was analyzed assessing the size and the composition. Micrographing and energy dispersive spectroscopic analysis of the dust were carried out while X-Ray diffraction (D8 Advanced diffractometer) with CuKα radiation was conducted analyzing the dust compounds. The particle adhesion on hydrophobized sample surface was measured using atomic force microscope.

## Results and Discussion

Droplet behavior on soft hydrophobic surface is considered. Polyisoprene elastomer is incorporated in the experiments and the sample surfaces are hydrophobized. Deposition of nano-size silica particles is realized towards hydrophobizing the sample surfaces.

### Hydrophobized surface and dust characteristics

**Figure 1:**
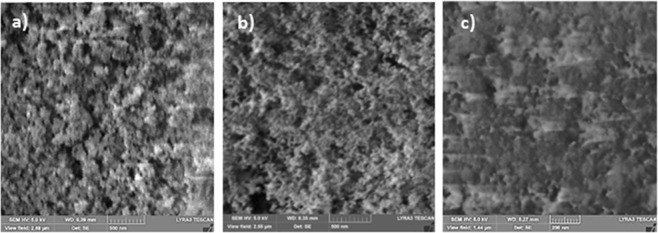
SEM micrographs of nano-size functionalized silica particles: (**a**) unstretched surface, (**b**) stretched surface, and (**c**) stretched relax surface.

**Figure 2:**
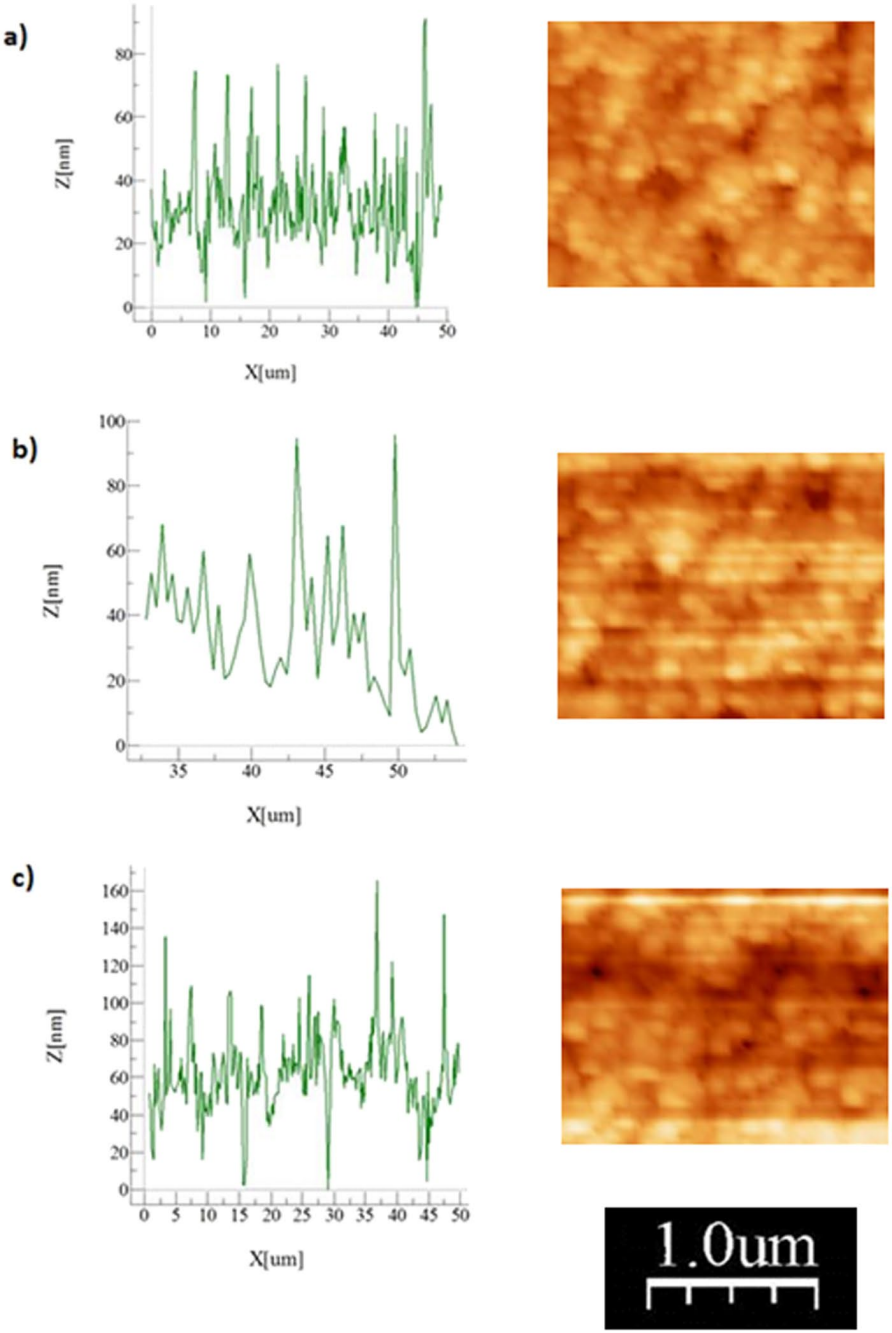
AFM line scan and surface image of nano-size functionalized silica particles: (**a**) unstretched surface, (**b**) stretched surface, and (**c**) stretched relax surface.

Figure 1a depicts micro-image of the hydrophobized surface obtained from SEM. The hydrophobized polyisoprene elastomer surface composes of closely located nano-size silica particles. The particles are about 30 nm in diameter and some small porous-like textures are also formed among the particles. The free energy of the hydrophobized surface is measured incorporating the contact angle technique using water, glycerol, and ethylene glycol^27,28^. The surface free energy is found to be about 35.51 mJ/m2, which contributes to hydrophobicity of the coated surface. The wetting state of the nano-size particles coated surface is determined using the goniometer. The surface contact angle is measured as 135° ± 3° and contact angle hysteresis of 3° ± 1°. Consequently, small hysteresis allows liquid droplet rolls on the coated polyisoprene elastomer surface. polyisoprene elastomer is stretchable material and coated samples are stretched and resulting surface texture topology and wetting states are analyzed. Samples are stretched 50% of its original size and the changes in the surface topology are recorded using SEM. Figure 1b depicts SEM micrograph of stretched surface. The clustered structures of the nano-size silica particles are observed. Hence, stretching of the sample does not cause disintegration of texture topology; in this case, the spacing between the nano-silica particles becomes slightly different than that of shown in Fig. 1a. The water contact angle also reduces to 122° ± 3° and contact angle hysteresis increases to 6° ± 1°. However, as the stretched sample is relaxed, it returns to original structure state and the closely agglomerated nano-size silica particles are observed (Fig. 1c). For the relaxed samples, the water droplet contact angle increases to 132° ± 3° with the hysteresis of 5° ± 1°. Hence, stretching and relaxing (unstretching) of samples result in reversible exchange of the texture topology and the wetting state of the surface. Moreover, the process of coating of nano-size silica particles is applied to the plain glass surfaces for comparison reason. The droplet contact angle of coated glass surface reaches at 150 ° ± 1° with contact angle hysteresis of 2° ± 1°. Hence, changing the sample from polyisoprene elastomer to glass increases the droplet contact angle significantly. Figure 2 depicts the atomic force microscopy line scan data and surface image for coated (Fig. 2a), coated and stretched (Fig. 2b), and coated and relaxed (Fig. 2c) sample surfaces. The texture height extends 70 nm and the porous-like texture is about 4 to 6 µm apart; consequently, agglomeration of the deposited nano-particles forms closely spaced texture morphology on the surface. The rippling along the line scan height in Fig. 2a demonstrates the closely located deposited nano-size particles on the surface. The roughness parameter of the coated, stretched, and unstretched surface is assessed via AFM surface images. The roughness parameter is determined about 0.52 for unstretched surface while it is about 0.49 for stretched surface and 0.52 for the relaxed surface. The roughness parameter is determined from the ratio of area of pillars over the project area^29^. Consequently, a small change in the roughness parameter results in almost 10° variation of the droplet contact angle on the sample surface.

**Figure 3:**
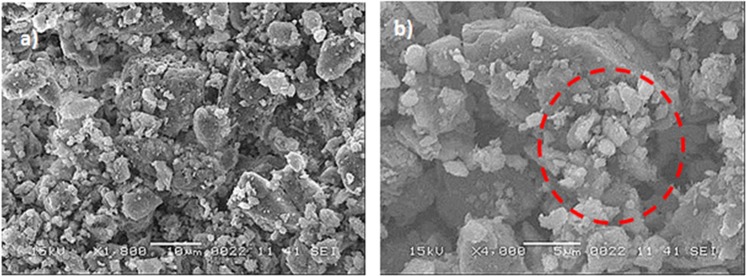
SEM micrographs of dust particles: (**a**) various size dust, and (**b**) clustered small dust (in circle).

**Figure 4:**
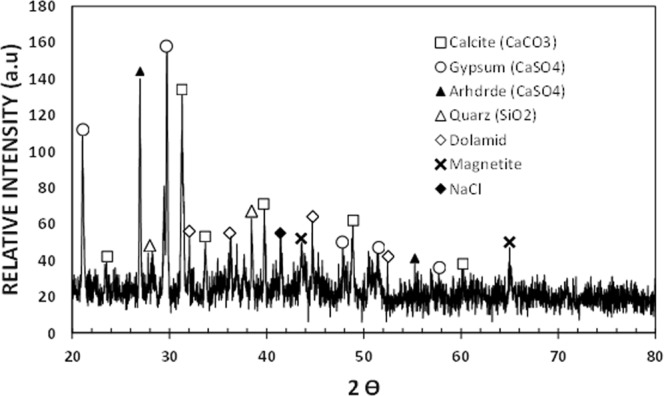
X-ray diffraction of dust particles.

Figure 3 shows SEM dust images. The dust size and shape vary considerably (Fig. 3a) and the average dust size is obtained from the particle size analyzer (utilizing Microtrac Nanotrac Wave Nanotechnology Particle Size Analyzer), which is about 1.2 µm. Small dusts adhere to the large dust surfaces and they agglomerate to form cluster like assemblies (Fig. 3b). The bright appearance of the small dust particles in SEM micrograph demonstrates that these particles are charged, which may be associated with the long exposure duration of sun radiation in atmosphere^30^. The chemical configuration of the dust is given in Table 2. The elemental composition reveals the occurrence of various elements inside the dust. However, depending on the dust size, elemental composition varies slightly and small particles contain high oxygen content (Table 2). Figure 4 shows x-Ray diffraction results of the dust. The peaks of the diffractogram shows the presence of the various compounds in the particle. The potassium and sodium peaks are due to alkaline/alkaline compounds in the particles, which can be related to the salt, since the dust are collected in Dammam area being closed to the Gulf Sea. Sulphur in the diffractogram is attributed to calcium in the form of anhydrite or gypsum (CaSO_4_). In addition, iron is probably because of clay-aggregated hematite (Fe_2_O_3_). The surface energy of the dust is assessed forming the pellets from the dust particles. In this case, the dust particles are compressed to form 20 mm diameter and 5 mm thick pellets. The excessive pressing of the dust is avoided during forming the pellets. The droplet contact angle technique is adopted to measure the surface free energy of the dust in accordance with the early work^23,24^. In this case, water, glycerol, and ethylene glycol are used in the measurements. Experiments are repeated seven times to ensure the repeatability of the data. The error related to the measurement is estimated about 7% and the surface free energy of the dust pellets is determined as 112.2 ± 5.2 mJ/m^2^. The dust adhesion on the uncoated and coated surfaces is assessed through the tangential force measurement by an atomic force microscope. The atomic force microscope probe in friction mode undergoes a strain when the probe opposes the dust particle. The probe strain results in probe deflection and the slope of the deflection can be related to the force generated on the probe tip according to $F=k\sigma \Delta V$^31^. Here *k* corresponds to the spring constant of the probe tip (N/m), σ is the slope of the deflection (Δz/ΔV, which is m/V), and ΔV represents the probe voltage (mV) obtained in the course of probe tip scanning (contact-mode). The probe has a linear spring-slope constant of *kσ* = 0.960908*nN*/*mV*. The deflection voltage measured is about 20.5 mV for coated surface and the resulting force for removal of 1.1 µm size dust particle on the coated surface is about 19.6986 nN. However, similar measurement is carried out for same size dust particles located on the uncoated sample surface. The findings reveal that the adhesion force determined from the atomic force microscope data is about 153,5824 nN, which is almost eight times of that corresponding to the coated surface. Experiments are repeated seven times to obtain the accurate results and the error estimated from the measurement is about 11%. Nevertheless, the dust particles adhere on to the uncoated surface stronger than that of the coated surface. Hence, the coating of nano-size silica particles lowers significantly the dust adhesion on the surface. The dust adhesion tests are repeated for the stretched and stretched relaxed coated surfaces. The stretching increased the adhesion force to 21.1165 nN, which is almost 7% of the adhesion force increased as compared to unstretched coated surface. On the other hand, adhesion force is about 19.9912 nN for the stretched relaxed coated surface; hence, the adhesion force increased less than 2%. Consequently, the force needed to remove the dust increases slightly for the stretched coated surface and it almost remain same for the stretched relaxed coated surface in reference to the coated unstretched elastomer surface.

**Table 2:** Elemental composition of dust (wt.%) determined by energy dispersive spectroscopy (EDS).

	Si	Ca	Na	S	Mg	K	Fe	Cl	O
Size ≥ 1.2 μm	11.4	8.5	2.1	1.3	2.1	0.9	1.1	0.4	Balance
Size < 1.2 μm	10.4	7.4	2.3	2.2	1.4	1.2	1.1	0.9	Balance

### Droplet-rolling-sliding on stretchable surface

**Figure 5:**
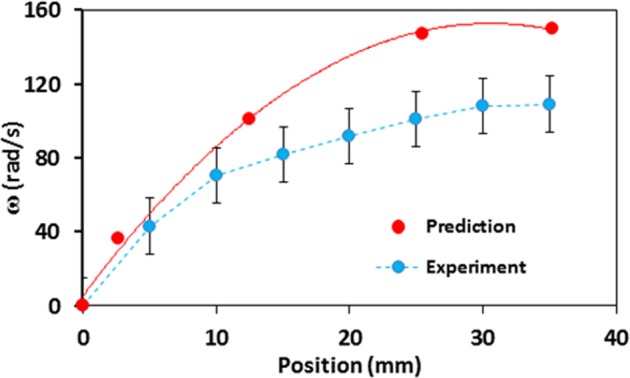
Rotational velocity (ω) obtained from Eq. 1 and experiment.

The droplets can roll/slide under the gravitational force (≥ 1.85 × 10^−4^ N). The droplet rotational speed (*ω*) is formulated previously incorporating the consideration of forces acting on a droplet, which is located on the hydrophobic surface^23^. The resulting equation yields:

1 $$\omega =\sqrt{(\frac{\frac{5}{2mR}(mg\,\sin \,\delta -\frac{24}{{\pi }^{3}}\sigma f(\cos \,{\theta }_{R}-\,\cos \,{\theta }_{A})\,-{\mu }_{t}{A}_{w}\frac{\partial u}{\partial y}-{\mu }_{f}mg)}{1+\frac{5}{4m}{C}_{d}{\rho }_{a}{A}_{c}R})}$$

where *m* represents mass of the droplet, *R* corresponds to radius of the spherical droplet (prior formation on surface), *g* is the gravitational acceleration, *δ* is inclination angle of hydrophobic surface, *γ* is surface tension, *f* is roughness parameter, *θ*_*R*_ is droplet receding angle, *θ*_*A*_ is droplet advancing angle, *μ*_*t*_ is fluid viscosity, *A*_*w*_ is droplet wetted area on hydrophobic surface, $\frac{\partial u}{\partial y}$ is rate of fluid strain, *µ*_*f*_ is friction coefficient, *C*_*d*_ is drag coefficient, *ρ*_*a*_ is air density, and *A*_*c*_ droplet cross-sectional area. Equation 1 includes all the major forces acting on the droplet such as inertial, pinning, shear, frictional, and drag forces. Figure 5 depicts the droplet rotational speed on the unstretched hydrophobic surface determined using Eq. 1 and that obtained experimentally from the high-speed records. The prediction and experimental data correspond to droplet volume of 40 µL and the surface inclination angle of 10°. The receding angle (*θ*_*R*_) varies within 118° – 121° depending on the location of the droplet on the inclined surface. The advancing angle (*θ*_*A*_) varies within 114° to 134° during droplet rolling on the inclines surface. The roughness parameter (*f*), which is obtained from AFM data, is about 0.52, surface tension (*σ*) and viscosity of water (*µ*_*t*_) are 0.073 N/m and 8.90 × 10^−4^ N/m^2^.s, respectively. The wetted diameter of the droplet on the hydrophobic surface is in the order of 2.72 mm for 40 μL droplet and it varies slightly along the inclined surface depending on droplet pinning. The predictions are in agreement with those resulted from the experiments. Increasing distance along the surface results in droplet rotational acceleration. On the other hand, the puddling effect is observed during the droplet rolling because of the gravitational influence. However, for the droplet diameter less than the capillary length (${\kappa }^{-1}=\sqrt{\frac{\gamma }{\rho g}}$, where *k*^−1^ corresponds to the capillarity length, *γ* represents the surface tension, and *ρ* is the density) remains in the spherical form during rolling^23^. The puddle height of the droplet is expressed earlier^32^ in the form of $\sqrt{2(1-cos\theta )\frac{\gamma }{\rho g}}$, here *θ* represents the contact angle, which demonstrates the dependence of the puddle height on the contact angle and capillary length. Moreover, the mass center of the droplet changes during the puddling and it deviates a small distance (*λ*) from the initial droplet mass center. It is demonstrated that the potential energy difference between the puddled and spherical droplets is expressed approximately as: $\cong \frac{\rho g{R}^{3}}{\gamma }$, here *R* represents radius of the droplet and *γ* corresponds to the surface tension^33^. The wetting length of the droplet on the hydrophobic surface can be written as: $l\cong \sqrt{R\lambda }$ during the puddling. Hence, the wetted length of the droplet undergoing puddling can be expressed as: $l\cong {R}^{2}/\sqrt{\frac{\gamma }{\rho g}}$, which is similar to that reported in the early study^33^. Moreover, the gravitational energy minimization, due to deforming of spherical droplets under puddling, yields the relation in terms of the wetting length, i.e.: $\rho g{R}^{3}\lambda  \sim \gamma {l}^{4}/{R}^{2}$. This demonstrates that increasing droplet wetted length causes the large shift in the mass center difference (*λ*), i.e. increased droplet puddling on the hydrophobic surface. During one rotational cycle of the droplet (rolling cycle), the shift in the mass center is estimated as 0.38 mm for a 3.15 mm droplet diameter. This finding agrees well with that of the formulation ($\lambda \cong \frac{\rho g{R}^{3}}{\gamma }$). The droplet puddling causes the wobbling of the droplet on the hydrophobic surface during the rolling. This causes variation of the droplet maximum height during the rolling. Moreover, the pinning force resulted because of the droplet adhesion under surface tension influence (${F}_{ad}=\frac{24}{{\pi }^{3}}{\gamma }_{LV}fD(cos{\theta }_{R}-cos{\theta }_{A})$, here, *f* corresponds to the texture solid fraction, *D* corresponds to the equivalent droplet diameter when it is spherical, ${\theta }_{R}$ represents the angle of receding, and ${\theta }_{A}$ is the angle of advancing.) causes sliding of droplet as it rolls. The translational droplet velocity, including the combination of rolling and sliding, can be formulated incorporating the energy balance of the droplet^23^. Hence, the droplet translational velocity along the hydrophobic surface is^4^:

2 $$V=\sqrt{\begin{array}{c}2g[\Delta Lsin\delta -{\mu }_{f}\Delta L-\frac{1}{mg}\frac{24}{{\pi }^{3}}\gamma Df\Delta L(cos{\theta }_{R}-cos{\theta }_{A})\\ -\frac{4\gamma }{\rho g\Delta L}(\frac{{D}_{{h}_{1}-}{D}_{{h}_{2}}}{{D}_{{h}_{1}}{D}_{{h}_{2}}})-\frac{1}{mg}{A}_{w}({\mu }_{t}\frac{d{V}_{f}}{dy})\Delta L-\frac{1}{2g}{K}_{L}{U}_{T}^{2}]\end{array}}$$

where ${D}_{{h}_{1}}$ and ${D}_{{h}_{2}}$ correspond to the droplet hydraulic diameter within the length scale *ΔL* on the surface and they are associated with the deformed volume of the droplet as it is rolling and puddling. *K*_*L*_ corresponds to the loss coefficient due to air resistance, and *U*_*T*_ represents the velocity of air flow around the droplet during rolling, which is assumed to be same order of the droplet translational velocity (*V*). *V*_*f*_ is the flow velocity inside the droplet and *y* is the distance, which is normal to the hydrophobic surface. The scale analysis is carried out to assess the influencing terms on the translational velocity in Eq. 2, the gravitational term ($\Delta Lsin\delta$), pinning term ($\frac{1}{mg}\frac{24}{{\pi }^{3}}\gamma Df\Delta L(cos{\theta }_{R}-cos{\theta }_{A})$), and the term involved with the work done during wobbling ($\frac{4\gamma }{\rho g\Delta L}(\frac{{D}_{{h}_{1}-}{D}_{{h}_{2}}}{{D}_{{h}_{1}}{D}_{{h}_{2}}})$) are the most effective terms on the magnitude of the translational velocity.

**Figure 6:**
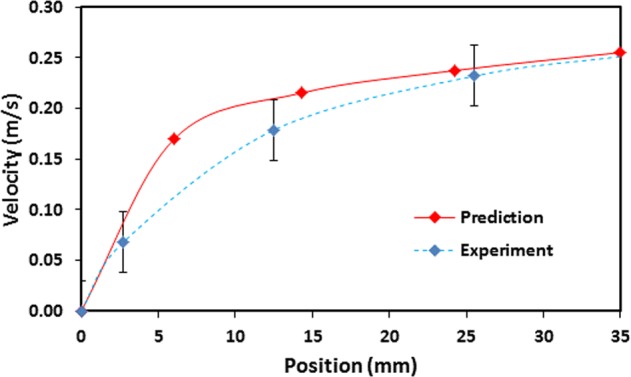
Droplet translational velocity predicted and measured.

Figure 6 shows translational velocity predicted from Eq. 2 and measured incorporating the data obtained from the experiment. The predictions and experimental findings in Fig. 6 correspond to the droplet volume of 40 µL and the surface inclination angle of 10°. Translational velocity increases with increasing length scale along the surface. However, non-linear velocity increase is related to the pinning force and gravitational energy loss due to work done during the droplet wobbling. The prediction of the translational velocity agrees with that corresponding to the measurements.

**Figure 7:**
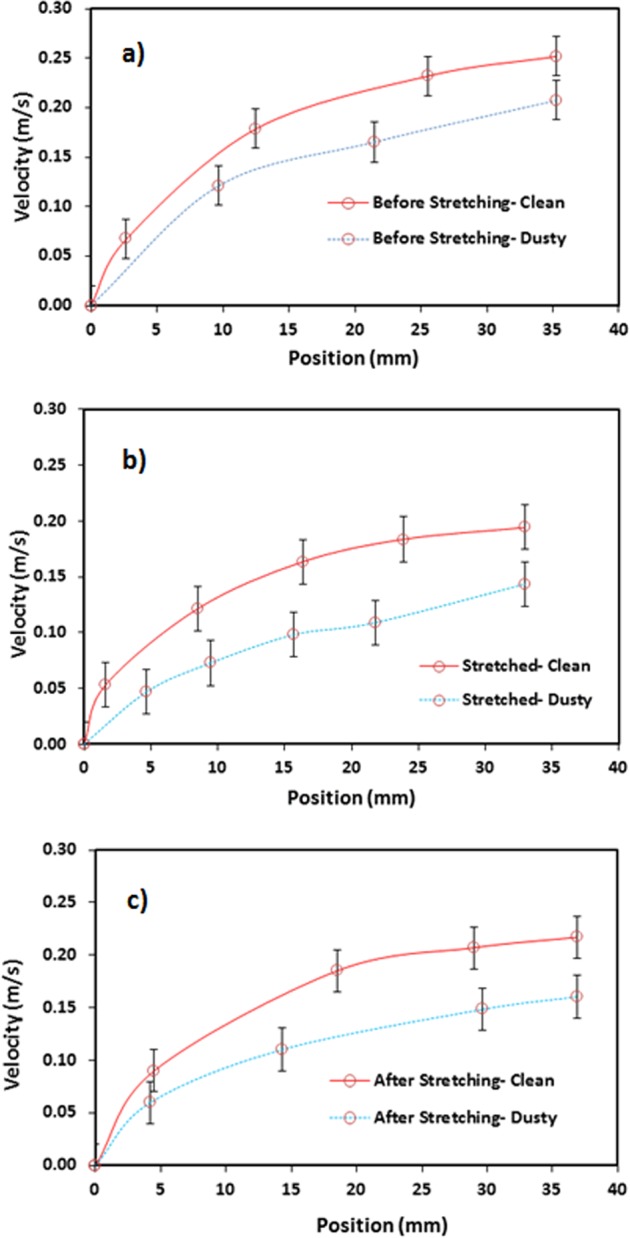
Translational velocity of droplet along hydrophobic surface: (**a**) unstretched, (**b**) stretched, and (**c**) relaxed surfaces.

**Figure 8:**
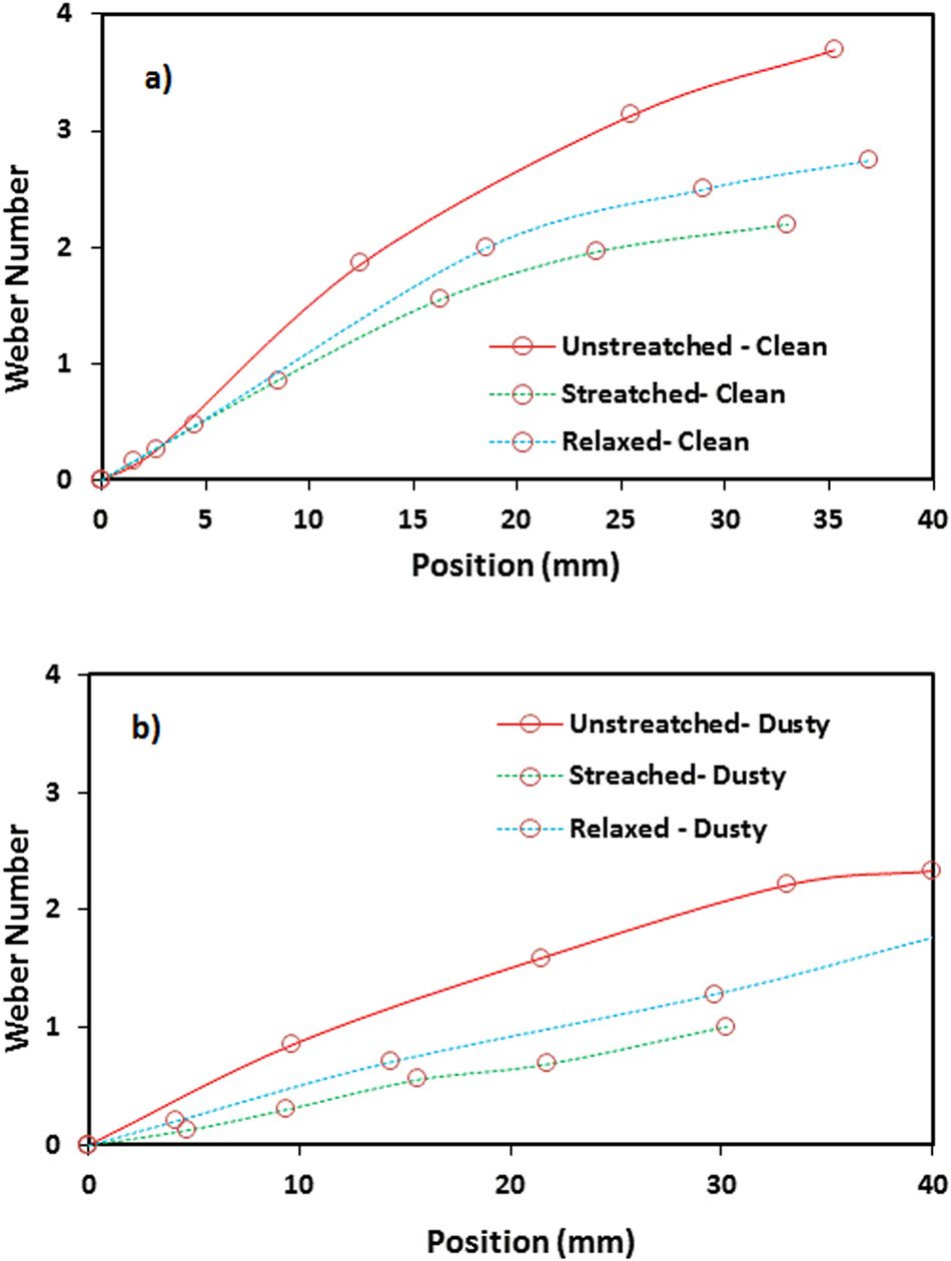
Rotational Weber number along hydrophobic surface for unstretched, stretched, and relaxed surfaces.: (**a**) clean (no dust), and (**b**) dusty surfaces.

For the dusty hydrophobic surface, the droplet behavior towards rolling and sliding changes. Figure 7 shows the droplet translational velocity obtained from the experiments for the cases with and without dust particles on unstretched, stretched, and relaxed hydrophobic surfaces. It should be noted that stretching experiments are repeated 12 times to ensure and build the confidence level for the repeatability of the data. In this case, the coated samples are stretched several times and the droplet rolling experiments are repeated on the resulting stretched surfaces. The findings show that the experimental error based on the several stretching of the surfaces is about 4%. Consequently, the stretched and stretched relaxed surfaces demonstrate reversible wetting state on the coated surfaces. Moreover, the droplet translational velocity enhances with the distance on the surface. The unstretched clean (without dust) sample results in higher velocity than those of the relaxed and stretched samples. This behavior is related to the wetting state of the unstretched surface (θ = 135° ± 3° and hysteresis is 3° ± 1°), which is higher than those of the stretched (θ = 122° ± 3° and hysteresis is 6° ± 1°) and relaxed (θ = 132° ± 3° and hysteresis is 5° ± 1°) surfaces. The contact angle and hysteresis alters the droplet motion on the surface. As the dust is placed on the sample surfaces, the droplet translational velocity becomes small, which is more pronounced for the stretched sample. The dust presence on the surface acts like a retarding site for the rolling and the sliding droplet while lowering its velocity. The gap in the droplet velocities because of clean and dusty surfaces remains large for the stretched surface. Hence, increasing hysteresis further lowers the droplet motion once the dust is deposited on the surface. Moreover, the rotational Bond number ($\frac{\rho {\omega }^{2}{R}^{3}}{8\gamma }$, where *ω* represents the angular velocity) is related to the ratio of centripetal over the surface tension forces. Hence, the dust presence lowers the angular velocity and the rotational Bond number. This can be observed from Fig. 8, in which rotational Bond number is given. In addition, droplet puddle on the surface during rolling influence the Bond number because of change of the location of the mass center of the droplet^23^.

**Figure 9:**
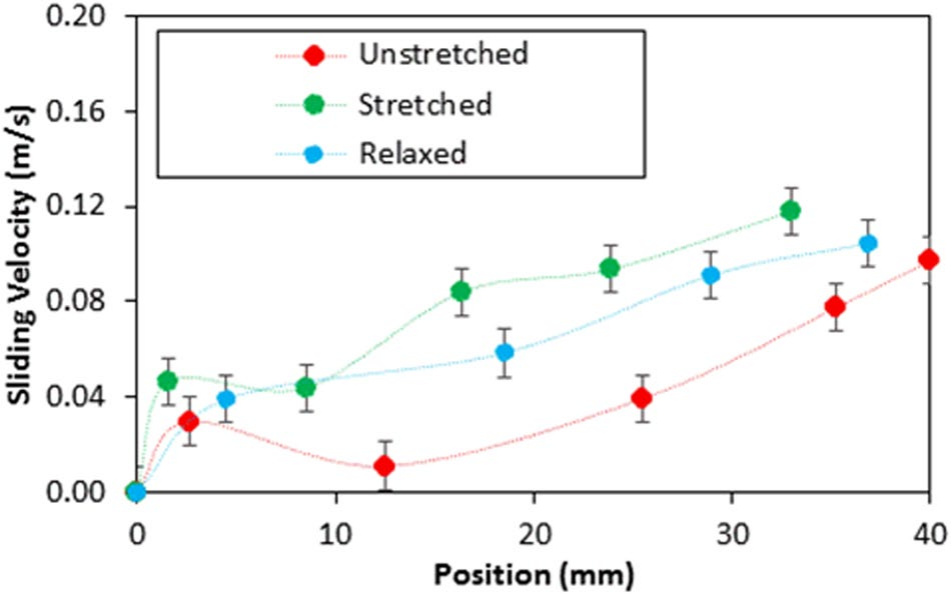
Sliding velocity of droplet on hydrophobic surface for unstretched, stretched, and relaxed surfaces.

**Figure 10:**
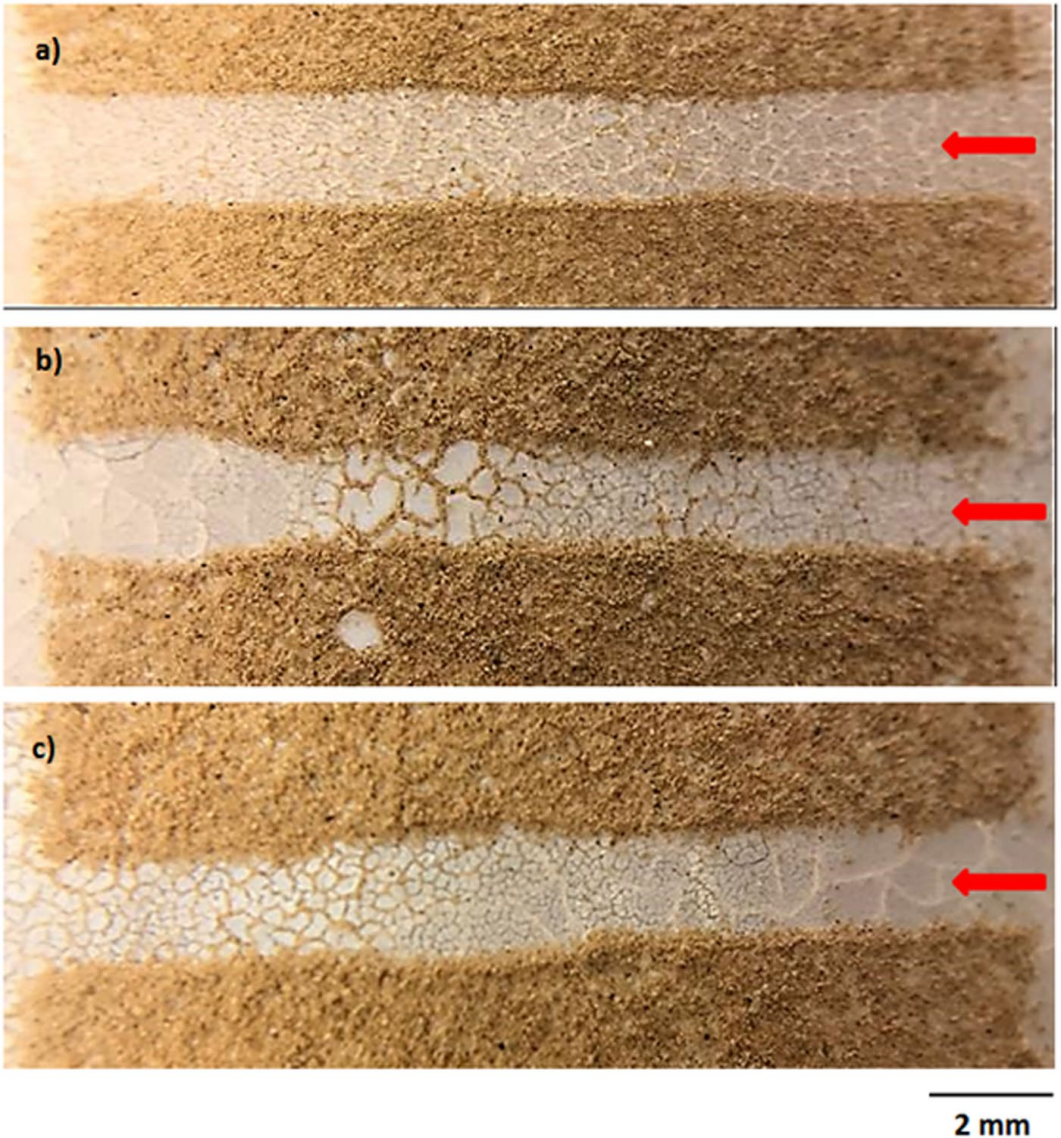
Optical images of droplet path: (**a**) unstretched, (**b**) stretched (circle shows the striations like structures on the edges), and (**c**) relaxed surfaces. Arrow shows droplet direction on dusty hydrophobic surface.

**Figure 11:**
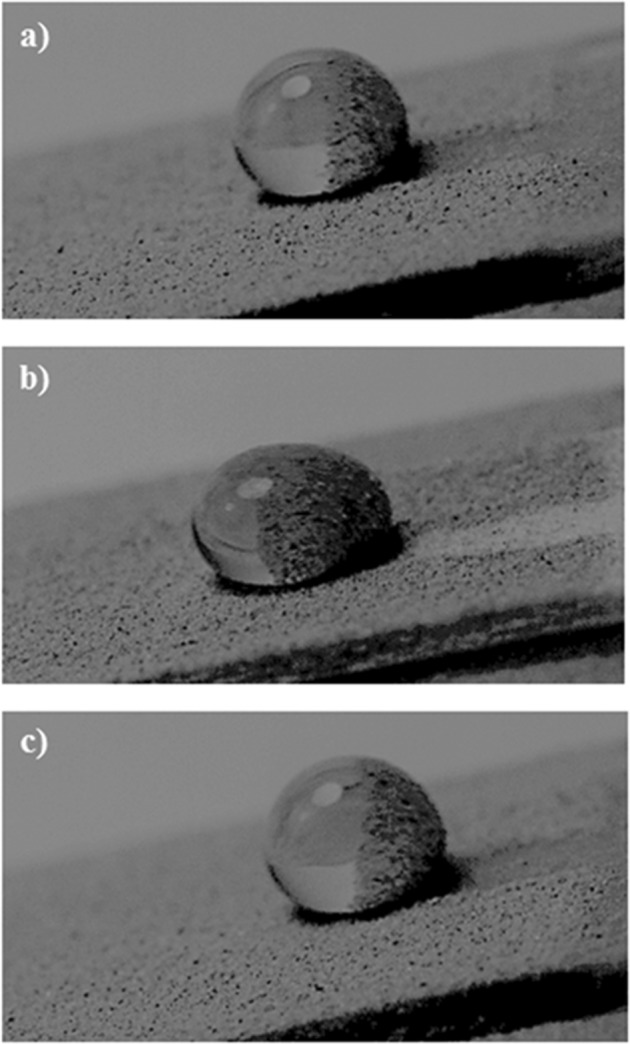
High speed data images for droplet on dusty surface: (**a**) unstretched surface, (**b**) stretched surface, and (**c**) relaxed surface.

**Figure 12:**
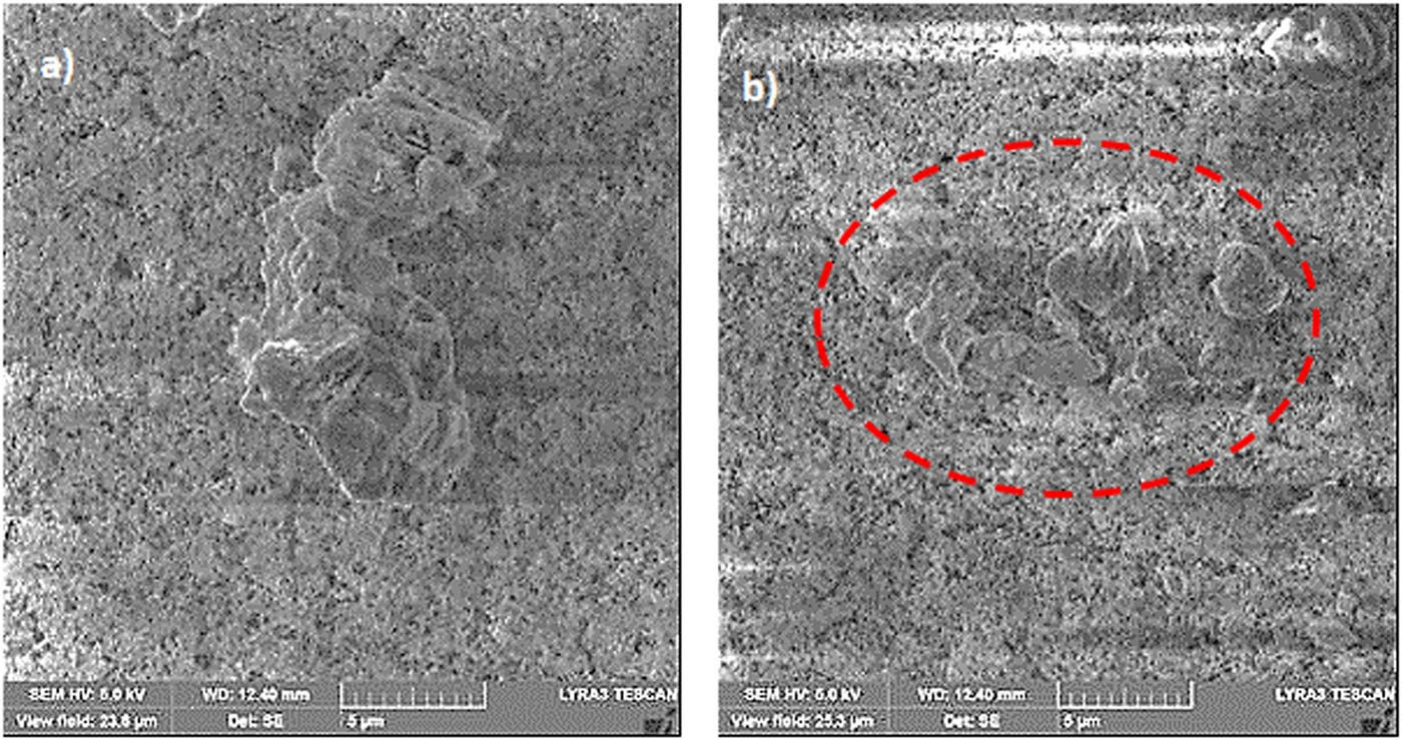
SEM micrographs of dust residues: (**a**) unstretched, (**b**) and stretched surfaces.

This alters the ratio between the rotational and translational velocities (ωR/V), which is consistent with the early findings^34^. It can be noted that the rotational speed is associated with the droplet fluid inertia during the rolling^23^ and the differences in the translational and rotational velocities of the droplet become same as the droplet sliding velocity. The puddling of the droplet also influences the dynamic pressure change in the fluid; hence, the dynamic pressure change is associated with the parameter $\phi =\frac{\Delta \rho {\omega }^{2}{R}^{2}}{{\rho }_{a}{V}^{2}}$, here Δρ represents the density difference in the droplet liquid and the air and *ρ*_*a*_ corresponds to the density of surrounding air^34^. The simulation data demonstrate that *φ* takes the values in the range of 750 to 900 depending on the volume of the droplet, which results in $\phi =\frac{\Delta \rho {\omega }^{2}{R}^{2}}{{\rho }_{a}{V}^{2}}\gg 1$; therefore, the droplet puddle does not considerably effect the dynamic pressure variation in the droplet as in agreement with the previously reported data^34^. Figure 9 depicts the droplet sliding velocity on the hydrophobic surface for the cases of unstretched, stretched, and relaxed sample surfaces. The sliding velocity changes with distance on the surface, which is related to the retarding forces, such as pining, shear, and frictional/form drag forces, generated on the surface, which causes puddling effect on the rolling droplet. Hence, the stage of the puddling alters on the surface while creating a varying sliding velocity of the droplet on the sample surfaces. The droplet sliding velocity increases with the distance. This indicates that the influence of the retarding force is increased and puddle effect becomes more pronounced as the droplet translational velocities increase (Fig. 8). When comparing the droplet sliding velocity on the unstretched, stretched, and relaxed surfaces, the sliding velocity attains larger values for stretched sample surface than that of the unstretched and relaxed sample surfaces. Although the rotational and translational velocities attain low values for the stretched surface due to the wetting state (low contact angle and high hysteresis), the difference between the translation and the rotational velocity increases. This, in turn, enhances the droplet sliding velocity on the surface. To estimate the dust removal and residues remaining along the droplet path along the surface, the droplet path optical records for unstretched, stretched, and relaxed surfaces are obtained. Figure 10 shows optical images of the droplet path for unstretched, stretched, and relaxed surfaces. The dust residues do not significantly seen within the droplet path, particularly for unstretached and relaxed surfaces. The striation like behavior along the droplet path is observed, which is attributed to droplet wobbling, i.e. the difference in the maximum-minimum widths of the path changes along the surface. This is more pronounced for stretched surface; hence, the increased droplet puddle on stretched surface is responsible for the increased striation like pattern along the path. Figure 11 shows high speed data for a water droplet on the dusty surface of unstretched, stretched, and relaxed samples. Droplet wetting length changes on the surface; hence, stretched sample demonstrates larger wetting length than those corresponding to unstretched and relaxed samples. The increasing wetting length reduces the droplet speed on the surface, which is also observed from Fig. 7. Moreover, the droplet removes the dust particles from the surfaces of all samples during its transition. Figure 12 shows SEM micrographs obtained from the droplet path on the unstretched, stretched, and relaxed surfaces. Only few dust residues are observed on unstretched surface (Fig. 12a) and the dust residues have small sizes. However, small cluster of the dust is observed on the stretched surface (Fig. 12b). The appearance of small dust clusters is attributed to the relatively smaller contact angle and larger hysteresis of the stretched surface as compared that of unstretched and relaxed samples. Nevertheless, almost 95% of the dust is removed along the droplet path by the droplet; hence, the droplet rolling and sliding provides cleaning of surfaces regardless of the state of surface such as stretched, unstretched, and relaxed.

## Conclusion

Water droplet motion on stretchable and hydrophobic elastomer surface is investigated in line with the self-cleaning applications. The properties of the environmental dust are investigated and the dust particle pinning on stretchable surface is evaluated using atomic force microscope. The droplet motion, combination of rolling and sliding, are recorded using a high speed recording system and resulting translational, rotational, and sliding velocities are analyzed for the cases of dust free and dusty surfaces. Stretchable polyisoprene elastomer sample surfaces are hydrophobized via depositing functionalized nano-size silica particles. It is found that hydrophobized polyisoprene elastomer surface results in droplet contact angle of 135° ± 3° and hysteresis is 3° ± 1°. As the hydrophobized sample is stretched by 50% in length, the wetting state of the surface alters with the contact angle of 122° ± 3° and hysteresis of 6° ± 1°. After relaxing the hydrophobized stretched surface the contact angle reversible increases to 132° ± 3° and hysteresis is about 5° ± 1°. The environmental dust constitutes several elements and elemental structure slightly changes for small size particles. The droplet translational velocity increases on the surface, which is more pronounced for unstretched sample surface. Relaxing of the stretched surface slightly changes the droplet translational velocity because of slightly large hysteresis of the relaxed sample. This is also observed for the droplet rotational velocity. Inclusion of the dust on hydrophobized surface lowers the droplet translational and rotational velocities. In this case, the dust acts like a retarding site for the rolling and sliding droplet while reduces the velocity. The droplet puddling effects the droplet motion on the hydrophobized surface such that the droplet maximum height and the wetting diameter changes along the droplet path. This causes formation of striation like structures of dust in the droplet path. The droplet sliding velocity increases slightly for the stretched surface, which is attributed to relatively higher hysteresis than the unstretched and relaxed surfaces. Droplet rolling/sliding can clean the dusty surface almost 95% and few dust remains (residues) are observed, which are small in size. The present study gives insight into the reversible exchanging of the surface wetting states via stretching and relaxing of the hydrophobized elastomer surfaces and offers understanding of the self-cleaning process incorporating the stretchable hydrophobic surfaces.
